# Low electric charge loading in a sequencing batch electro-membrane bioreactor: influence of aeration intensity on treatment performance, biomass activity, and membrane fouling

**DOI:** 10.1007/s11356-026-37984-6

**Published:** 2026-07-01

**Authors:** Tiago José Belli, Emerson Souza, Caroline Rodrigues, André Aguiar Battistelli, Wanderli Leite, Miriam Cristina Santos Amaral, Flávio Rubens Lapolli

**Affiliations:** 1 Department of Civil Engineering, Santa Catarina State University (UDESC), Ibirama, SC Brazil; 2 Department of Sanitary and Environmental Engineering, Federal University of Santa Catarina (UFSC), Florianópolis, SC Brazil; 3 Department of Environmental and Civil Engineering, Federal University of Pernambuco (UFPE), Recife, PE Brazil; 4 Department of Sanitary and Environmental Engineering, Federal University of Minas Gerais (UFMG), Belo Horizonte, MG Brazil

**Keywords:** Membrane bioreactor, Electrocoagulation, Aeration intensity, Electric charge loading, Sequencing batch reactor

## Abstract

This study investigates the performance of a sequencing batch electro-membrane bioreactor (SB-EMBR) operated under low electric charge loading (39.9 mAh L⁻^1^), focusing on the effects of aeration intensity on treatment performance, biomass activity, and membrane fouling. The reactor was operated at a current density of 10 A m^−2^ and three specific aeration demand levels (SADₘ = 0.48, 0.24, and 0.12 m^3^ m⁻^2^ h⁻^1^). Organic matter and phosphorus removals remained consistently high (> 90% COD removal; TP < 1.0 mg L⁻^1^) regardless of aeration intensity. In contrast, ammonium removal efficiency declined from 99.5 to 74.7% as the SADₘ decreased from 0.48 to 0.12 m^3^ m⁻^2^ h⁻^1^. Batch assays revealed reduced activity of polyphosphate-accumulating organisms under oxygen-limited conditions. The pronounced decrease in the P-release/COD-uptake ratio from 0.172 to 0.0164 mol P mol⁻^1^ C indicates that TP removal at low dissolved oxygen became predominantly governed by chemical coagulation rather than the biological phosphorus removal process. The calculated Al/P molar ratio of 2.32 mol Al mol⁻^1^ P was sufficient to sustain phosphorus removal through both precipitation and adsorption onto aluminum hydroxides. Reduced aeration favored anoxic phosphorus uptake, increasing the denitrifying phosphate assimilation potential from 18 to 41%. The membrane fouling rate increased from 1.02 to 4.81 kPa d⁻^1^ as aeration decreased, mainly due to diminished shear forces, soluble microbial products accumulation (+ 205%), floc size reduction (− 50.1%), and higher capillary suction time (+ 123%). Owing to the short current application (1.6 h d⁻^1^), the additional electrocoagulation cost was only 0.07 USD m⁻^3^, lower than values reported for continuous-flow EMBRs. Overall, operation of the SB-EMBR under reduced electric charge loading demonstrated promising energy efficiency while maintaining stable and satisfactory pollutant removal even at low aeration intensities.

**Supplementary Information:**

The online version contains supplementary material available at 10.1007/s11356-026-37984-6.

## Introduction

Membrane bioreactors (MBRs) have become consolidated as a well-established technology for wastewater treatment. The systems constitute an advanced technological alternative that integrates biological treatment with membrane filtration processes, such as microfiltration or ultrafiltration. This combination offers several advantages, including high effluent quality, a compact footprint, low sludge production, and reliable removal of pathogenic microorganisms (He et al. 2024). Initially, MBRs were primarily implemented in small-scale wastewater treatment facilities, such as those serving the food industry or decentralized residential areas, with treatment capacities of less than a few thousand tons per day. However, since the early 2000s, their application has expanded significantly, with system capacities increasing from over 10,000 to several 100,000 tons per day (Kobayashi and Terada 2025).

Despite the successful application of MBRs for treating both municipal (Meng et al. 2017) and industrial wastewater (Zhang et al. 2021), ensuring stable permeate quality and filtration performance remains a critical challenge to support their large-scale adoption and long-term operational reliability. Therefore, membrane fouling represents a major operational concern in MBR systems (Sandoval-García et al. 2025). The accumulation of foulants during filtration increases the transmembrane pressure (TMP) and reduces membrane permeability, ultimately making chemical cleaning necessary to restore membrane performance.

Electrically-assisted strategies have recently been integrated into membrane bioreactors (MBRs) with the primary objective of mitigating membrane fouling and enhancing permeability (Meng et al. 2017). In the so-called electro-membrane bioreactor (EMBR), these improvements are accompanied not only by more effective fouling control but also by enhanced pollutant removal efficiency. The filterability of the mixed liquor in EMBR systems is improved through various electrochemical mechanisms, such as electrophoresis, electrostatic repulsion, electroflocculation, electrochemical oxidation, and electroosmosis, which collectively contribute to reducing membrane fouling potential (Zhang et al. 2023). In parallel, the metallic hydroxides generated by anode dissolution act as coagulants, promoting the neutralization and precipitation of suspended organic matter, colloidal particles, and PO_4_^3−^-P ions, thereby contributing to enhanced reactor treatment performance (Moussa et al. 2017; Ensano et al. 2019). Compared to conventional chemical coagulation, electrocoagulation integrated into MBRs offers several operational advantages, including reduced sludge production, elimination of chemical reagent dosing, and lower alkalinity consumption (Bani-Melhem et al. 2023). Although this integration may increase operational costs, the reduction in fouling rates and the corresponding decrease in chemical cleaning demand may offset the additional expenses(Asif et al. 2020). For instance, Xiao et al. (2019) estimated that membrane cleaning chemicals can account for 10–30% of the total operating cost of MBRs. Furthermore, electrocoagulation has been associated with improved permeate quality, which is particularly advantageous where water reuse is required.

The EMBRs systems has been applied to the treatment of a wide range of wastewater streams, including landfill leachate (El Hachimi et al. 2025), effluent from detergent manufacturing plants (Abdollahzadeh Sharghi et al. 2025), municipal wastewater (Chen et al. 2020), phenol wastewater (Sun et al. 2024), and pharmaceutical wastewater (Zare et al. 2024). Although interest in EMBRs has grown in recent years, research has predominantly focused on continuous-flow configurations, whereas studies involving sequencing batch operation remain scarce. Under sequencing batch conditions, intermittent filtration may mitigate membrane fouling by reducing solids accumulation on the membrane surface while maintaining aeration-induced shear during non-filtration stages (Zhang et al. 2006). To date, only Souza et al. (2020) have evaluated the sequencing batch electro-membrane bioreactor (SB-EMBR) configuration as alternative to wastewater treatment. While providing relevant insights into membrane fouling control, the study offered limited discussion on the pollutant removal mechanisms, particularly with respect to nitrogen and phosphorus dynamics under sequencing batch conditions. The effects of electrocoagulation on enhanced biological phosphorus removal (EBPR) and its potential interference with phosphate uptake by polyphosphate-accumulating organisms (PAOs) are still poorly understood. Given these knowledge gaps and the inherent operational flexibility of sequencing batch reactors (Pajoumshariati et al. 2017), further studies assessing the performance and applicability of SB-EMBRs for municipal wastewater treatment are essential.

In addition to these scientific gaps, EMBR systems may face practical limitations associated with energy consumption and electrode deterioration. Previous studies have commonly employed prolonged current application periods (5–10 h day⁻^1^) and high electric charge loadings (214–428 mAh L⁻^1^), resulting in increased energy demand, accelerated anodic corrosion, and more frequent electrode replacement (Asif et al. 2020; Mendes Predolin et al. 2021; Sharghi et al. 2024). Therefore, reducing electrochemical exposure time represents an important strategy to improve the energy efficiency and operational sustainability of EMBRs. Nevertheless, studies evaluating the long-term performance of EMBRs under low electric charge loading conditions remain scarce, particularly for sequencing batch configurations, in which intermittent operation may further attenuate anodic corrosion and extend electrode lifespan. Unlike previous studies primarily focused on fouling mitigation or continuous-flow operation, the present work specifically investigates the interactions among aeration intensity, biological nutrient removal, electrocoagulation-assisted phosphorus removal, and membrane fouling behavior under low electric charge loading conditions in an SB-EMBR system.

To address these aspects, this study systematically evaluates the effect of low exposure time (1.6 h day^−1^) and electric charge loading (39.9 mAh L⁻^1^) in an SB-EMBR on treatment performance (COD, N, P), biomass activity, and membrane fouling behavior. In parallel, the SB-EMBR was operated under reduced aeration intensity, applying specific aeration demand per membrane area (SADₘ) values ranging from 0.48 to 0.12 m^3^ m^−2^ h^−1^, all below the range typically reported for full-scale MBR systems (0.5–1.5 m^3^ m⁻^2^ h⁻^1^) (Judd 2006). In this context, the present study aimed to evaluate SB-EMBR performance under reduced electric charge loading and low aeration intensity (0.48, 0.24, and 0.12 m^3^ m^−2^ h^−1^). The investigation focused on the effects of aeration on nitrogen removal, EBPR process performance, and membrane fouling dynamics. Ammonium oxidation and phosphorus release/uptake behaviors were monitored throughout the aerated and non-aerated phases. Complementary batch activity tests were conducted to support the observations from cyclic operation. In addition, sludge characteristics were analyzed to elucidate fouling mechanisms. Finally, the energy demand associated with different SADₘ conditions and the operational costs related to electrocoagulation were estimated and compared with values reported for continuous-flow EMBR systems.

## Materials and methods

### Experimental setup and operating conditions

**Fig. 1:**
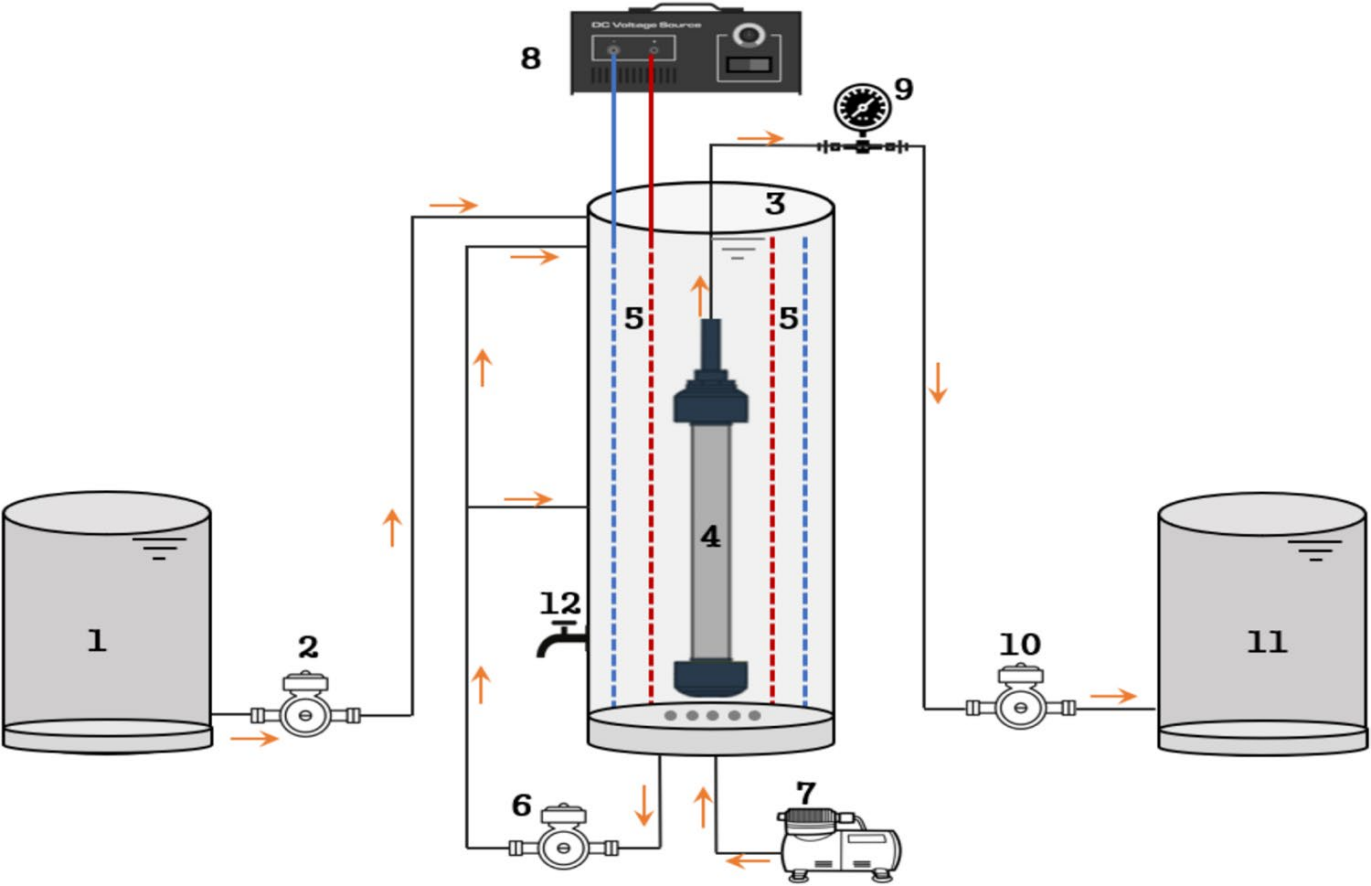
Experimental setup. (1) Influent. (2) Feeding pump. (3) EMBR. (4) Membrane. (5) Electrodes. (6) Recirculation pump. (7) Air blower. (8) DC power supply. (9) Pressure gauge. (10) Filtration pump. (11) Effluent. (12) Sludge sampling

A lab-scale SB-EMBR system was constructed as depicted in Fig. 1. The SB-EMBR comprised a cylindrical Plexiglas reactor (working volume of 54 L), in which the hollow fiber ultrafiltration membrane (ZeeWeed, ZW-10–0.93 m^2^, SUEZ Water Technologies & Solutions) were submerged. Metallic electrodes (stainless-steel cathode and aluminum sacrificial anode) were fixed in the SB-EMBR, immersed around the membrane module. A digital direct current (DC) power supply (Model PS 1001) was connected to the electrodes to provide the electric current density of 10 Am^−2^. In order to alleviate membrane fouling, minimize electrode passivation and supply air for the biological process, spargers were installed at the bottom of the SB-EMBR tank. A peristaltic pump (Watson Marlow, 523 s) was connected to the membrane module to promote the filtration process, using permeation flux of 17.5 L m^−2^ h^−1^ under intermittent mode (1 min OFF/9 min ON). Transmembrane pressure (TMP) values were continuously monitored using a digital vacuum gauge (VDR-920, Instrutherm).

The reactor was operated in sequential batch mode, with a cycle time of 180 min comprising four phases: (1) feeding (2.5 min); (2) anoxic/anaerobic (42.5 min); (3) aeration (70 min), and (4) aeration/filtration (65 min). The SB-EMBR operated with a volumetric exchange rate (VER) of 30% (16 L/cycle), resulting in a hydraulic retention time (HRT) of 10 h. In the feeding and anoxic/anaerobic phases, a centrifugal pump recirculated the mixed liquor from the bottom to the top of the reactor to ensure complete mixing conditions. Throughout the aeration/filtration phase (65 min), the electric current was intermittently applied in cycles of 6 min ON/30 min OFF, resulting in a daily electric current application time of 1.6 h. During this period, the reactor was exposed to a current density (CD) of 10 A m^−2^. The SB-EMBR operating cycle was automated by a programmable logic controller (PLC, Siemens), which regulated the intermittency of both the filtration process and the electric current application. The solids retention time (SRT) was maintained in 15 days, by daily sludge wasting at the end of the aerobic phase.

Throughout the operating period, the SB-EMBR was fed with synthetic wastewater, simulating municipal wastewater, prepared with the following composition (Hua et al. 2015): 600 mg L^−1^ of CH_3_COONa, 200 mg L^−1^ of NH_4_Cl, 230 mg L^−1^ of KH_2_PO_4_, 7.3 mg L^−1^ of CaCl_2_⋅2H_2_O, 10 mg L^−1^ of MgSO_4_⋅7H_2_O, 0.2 mg L^−1^ of CuSO4⋅5H2O, 2.15 mg L^−1^ of MnSO_4_⋅H_2_O, 0.5 mg L^−1^ of CoCl_2_⋅6H_2_O, 5.00 mg L^−1^ of FeSO_4_⋅H_2_O, and 2.2 mg L^−1^ of ZnSO_4_⋅7H_2_O.

### Inoculation and experimental periods

The SB-EMBR was inoculated with activated sludge collected from a municipal wastewater treatment plant operated in sequencing batch mode (Florianópolis, Brazil). The reactor underwent a 60-day biomass acclimatization period (data not shown). Following this startup phase, the SB-EMBR was monitored for 150 days, during which three experimental conditions were investigated, divided into three periods of 50 days (periods I, II, and III). Considering the SRT of 15 days adopted, each operational period exceeded three times the applied SRT, thereby ensuring steady-state conditions throughout the monitored period (Hwang et al. 2010; Holzem et al. 2018). The only difference between these three periods was the air flow rate applied to the SB-EMBR and, consequently, the corresponding SADm. For the periods I, II, and III, the SB-EMBR was subjected to the SADm of 0.48, 0.24, and 0.12 m^3^ m^−2^ h^−1^, respectively (Table 1). As result, the average dissolved oxygen in the end of the aerated phase were 6.8, 2.7, and 0.7 mg L^−1^ in periods I, II, and III, respectively.

**Table 1:** Operating conditions of SB-EBMR for each period and corresponding influent concentrations in terms of COD, NH_4_^+^–N, TN, and TP

	SAD_m_ (m^3^m^−2^ h^−1^)	DO in the non-aerated phase^1^ (mg L^−1^)	DO in the aerated phase^2^ (mg L^−1^)	COD (mg L^−1^)	NH_4_^+^–N (mg L^−1^)	TN (mg L^−1^)	PO_4_^3−^–P (mg L^−1^)
Period I	0.48	0.3 ± 0.04	6.8 ± 0.2	496 ± 19	45.2 ± 3.8	45.3 ± 3.8	5.1 ± 07
Period II	0.24	0.2 ± 0.01	2.7 ± 0.7	501 ± 15	47.1 ± 3.1	47.5 ± 3.9	5.4 ± 0.7
Period III	0.12	0.2 ± 0.01	0.7 ± 0.9	510 ± 48	46.1 ± 4.5	46.4 ± 4.1	5.3 ± 0.3

*SAD*_*m*_, specific aeration demand per membrane area; *DO*, dissolved oxygen; *COD*, chemical oxygen demand; *TN*, total nitrogen; *TP*, total phosphorus

^1^Average DO concentration at end of the anoxic phase

^2^Average DO concentration at the end of the aerobic/filtration phase

### Batch experiments to assessing the anoxic phosphate removal potential

To evaluate the phosphate removal capability under anoxic and aerobic conditions, batch experiments were conducted throughout the experimental period, following the methodology described by Wachtmeister et al. (1997). Sludge samples (2 L) were collected from the SB-EMBR at the end of the aerobic phase and transferred to sealed vessels to determine phosphate uptake and release rates. The vessels were spiked with sodium acetate (0.2 g L^−1^) and maintained under anaerobic conditions for 3.5 h to promote phosphate release. Subsequently, half of the sludge volume (1 L) was incubated under oxygen-saturated conditions (7 mg O_2_ L⁻^1^) to evaluate the aerobic phosphate uptake rate, while the remaining portion was maintained under anoxic conditions (20 mg NO_3_⁻-N L⁻^1^) without aeration to assess the anoxic phosphate uptake rate. At predetermined time intervals, aliquots were collected from both vessels and filtered through a 0.45-μm cellulose acetate membrane for subsequent analysis of PO_4_^3^⁻-P concentrations. Phosphate uptake rates (PUR) under aerobic and anoxic conditions were calculated by linear regression of the concentration profiles over time. Based on the estimated PUR values, the contribution of anoxic phosphate removal to the total phosphate uptake (aerobic + anoxic) was determined. These batch experiments were conducted during the final stage of each experimental period to ensure representative steady-state SB-EMBR conditions.

### Membrane fouling rate, anode corrosion, and energy consumption

The TMP values were used to calculate the membrane fouling rate (MFR) over operating days (ΔT), based on Eq. 1 (Millanar-Marfa et al. 2022).

1 $${M}{F}{R}=\frac{\Delta {T}{M}{P}}{\Delta T}$$

To complement the MFR data and further evaluate mixed liquor filterability, dead-end filtration tests were performed using an unstirred filtration cell (Sartorius, 250 mL) operated at a constant pressure of 200 kPa. Each test lasted 20 min, and a new flat-sheet membrane with a pore size of 0.2 μm was used for each experiment. The permeate flux data obtained during filtration were used to calculate the specific resistance to filtration (SRF) according to Eq. 2 (Pollice et al. 2008):

2 $$\mathrm{S}\mathrm{R}\mathrm{F}=\frac{\mathrm{2,000}.{A}^{2}\Delta P}{\mu .C}.\frac{{~}^{t}\!\left/ \!{~}_{V}\right.}{V}$$

where SRF is the specific resistance to filtration (m kg^−1^); Δ*P* is the pressure applied (kPa); *A* is the membrane surface area; *V* is the permeate volume; *t* is the filtration time (s); *μ* is the effluent viscosity (Pa.s), and *C* is the MLSS value (kg m^−3^). The dead-end filtration tests were conducted in triplicate during the final stage of each experimental period to ensure representative steady-state conditions.

Reversible (${R}_{r}$) and irreversible (${R}_{ir}$) fouling resistances were estimated from TMP values measured before and after membrane cleaning procedures using Darcy’s law. The intrinsic membrane resistance (${R}_{m}$) was determined from the clean water permeability test. ${R}_{r}$ corresponded to the resistance removed after cleaning, whereas ${R}_{ir}$ represented the residual resistance remaining after the cleaning procedure.

During the three experimental conditions, the aluminum anode was exposed to the electrolytic corrosion process, resulting in Al^3+^ ion dissolution in the mixed liquor. To estimate this anodic corrosion process, Eq. 3 was used (Li et al. 2018).

3 $$m=\frac{{M}_{w}}{{F}_{a}Z}TI$$

where *m* is the anodic corrosion rate (g Al^3+^ day^−1^); *M*_*w*_ is the molar mass of the aluminum (26.98 g mol^−1^); *F*_*a*_ is the Faraday’s constant (96.485 C mol^−1^); *Z* is the valence of the aluminum (+ 3); *T* is the time of electric current applied daily (1.6 h/day), and *I* is the amperage (3.19 A).

Considering the daily permeate flow, the anode corrosion rate (*m)* per volume of effluent (*W*) may be estimated as expressed in Eq. 4 (Hasan et al. 2014).

4 $$W=\frac{{M}_{w}}{{F}_{a}ZQ}TI$$

wherein *W* is the anode corrosion per permeate volume (g m^−3^) and *Q* is the permeate flow (m^3^ day^−1^).

Energy consumption is a critical economic factor in wastewater treatment systems, especially in electrochemically assisted MBRs. In this context, the energy demand associated with the electrocoagulation process was estimated using Eq. 5 (Larue et al. 2003):

5 $$\mathrm{E}\mathrm{C}=\frac{U.I.T}{Q}$$

wherein EC is the energy consumption per wastewater volume (kWh m^−3^), *I* is the electric current applied (A), *U* is the voltage used in the metallic electrodes (V), *T* is the time of electrocoagulation (h day^−1^), and *Q* is the wastewater flow rate (m^3^ day^−1^).

The additional operating cost (USD m⁻^3^) related to the electrocoagulation process was calculated based on Eqs. 3 and 4. This estimative, detailed in Eq. 6, takes into account both energy consumption (electricity) and the anodic corrosion rate (Udomkittayachai et al. 2021).

6 $$C=a\mathrm{E}\mathrm{C}+bW$$

where *C* represents the additional operating cost linked to electrocoagulation (USD m^−3^); $a$ is the price of electrical energy (USD kWh^−1^); EC is the energy consumption (kWh m^−3^); $b$ is the price of the aluminum anode (USD kg^−1^), and *W* is the anode corrosion per permeate volume (kg m^−3^). Considering the Brazilian market in April 2026, the unit costs of “*a*” and “*b*” used in Eq. 5 were 0.1104 USD KWh^−1^ and 2.26 USD kg^−1^, respectively.

Volumetric charge loading (mAh L⁻^1^) refers to the quantity of electric charge applied per unit volume of treated wastewater (Roy et al. 2020). Its estimation is based on the current supplied to the electrodes (mA), the duration of current application (T), and the influent flow rate (L day⁻^1^). This parameter was determined using Eq. 7, as proposed by Ravadelli et al. (Ravadelli et al. 2021).

7 $$q= \frac{I \times T}{Q}$$

where *q* is the volumetric charge loading (mAh L⁻^1^), *I* is the amperage (mA), *T* is the duration of electric current application (h day⁻^1^), and *Q* represents the permeate flow rate (L day⁻^1^).

During the three experimental periods, the SB-EMBR was operated under the same current density (10 A m^−2^) and electrochemical exposure time (1.6 h day^−1^). Consequently, the theoretical estimations of $m$, $W$, $\mathrm{E}\mathrm{C}$, $C$, and $q$ were representative for all operational periods, regardless of aeration intensity. These calculated values were subsequently used to support a comparative discussion between the SB-EMBR and other EMBR configurations.

### Analytical methods and statistical analyses

COD, NH_4_^+^–N, PO_4_^3−^–P, mixed liquor suspended solids (MLSS), and mixed liquor volatile suspended solids (MLVSS) were determined in triplicate according to the standard methods (APHA, 2023).

Mixed liquor characteristics were evaluated based on capillary suction time (CST), particle size distribution (PSD), supernatant turbidity (SNT), soluble microbial products (SMP), and extracellular polymeric substances (EPS). These fouling-related parameters were monitored twice a week to assess the filterability of the mixed liquor. SMP and EPS were assessed in terms of polysaccharides (PS) and protein (PN) by spectrophotometric analysis. SMP was obtained by filtering SB-EMBR mixed liquor samples in 0.45-μm acetate filter. PS and PN content were determined in triplicate according to Lowry et al. (1951) and DuBois et al. (1956), respectively. To assess the capillary suction time (CST) of the SB-EMBR sludge, a 5 mL sample of mixed liquor was collected from the reactor at the end of the aerobic phase and subjected to CST analysis (304 M CST, Triton). Particle size distribution (PSD) was analyzed using laser diffraction technology (Mastersizer 2000, Malvern instruments Ltd.). The supernatant turbidity measured at the end of the sludge volume index test, after 30 min of settling, was used as an indirect parameter to assess sludge flocculation ability (Fallah et al. 2010). CST, PSD, and supernatant turbidity analyses were conducted twice a week throughout each experimental period. Statistical analysis was performed using analysis of variance (ANOVA), adopting a significance level of 0.05 for all analyses.

## Results and discussion

### Chemical oxygen demand (COD) removal

**Fig. 2:**
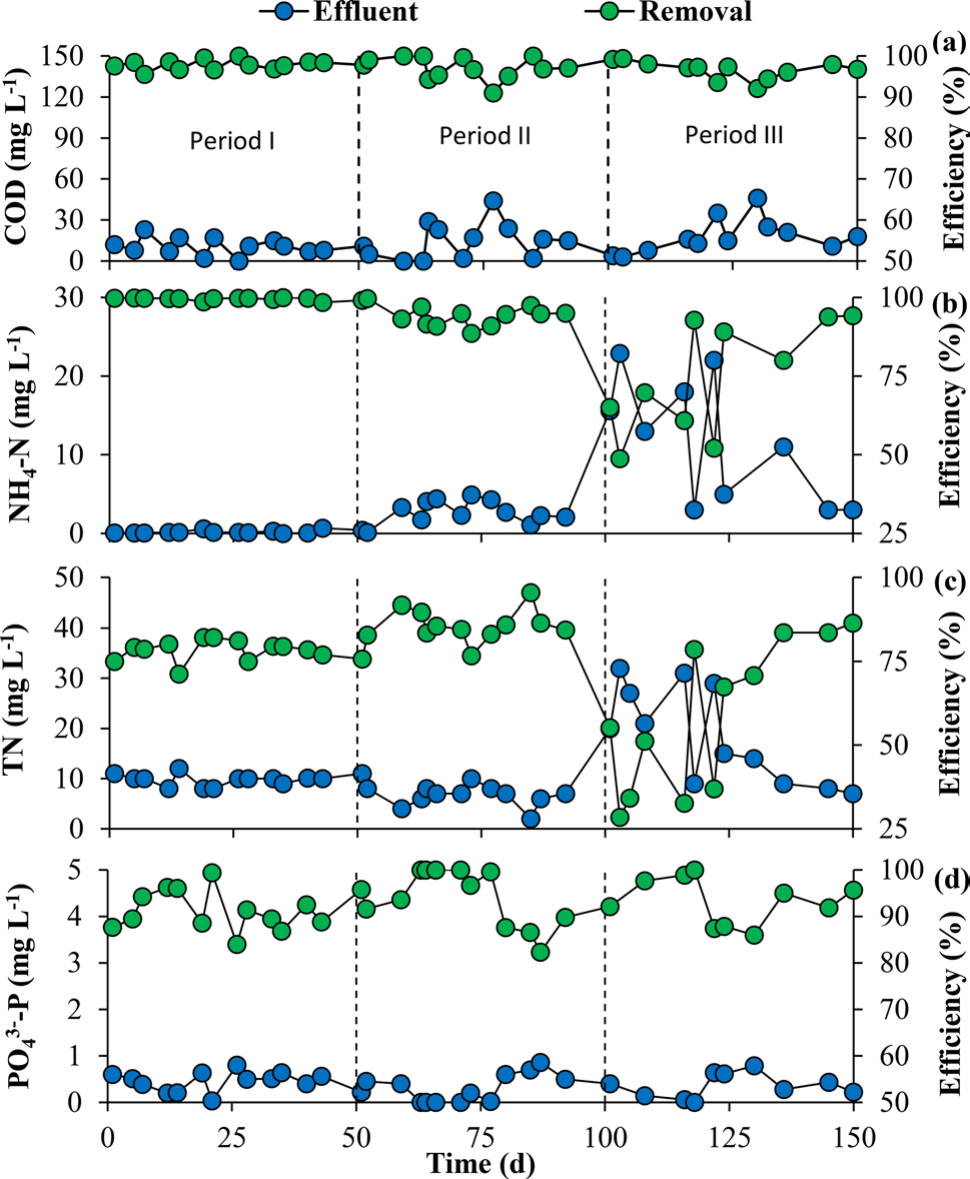
COD (**a**), NH_4_^+^–N (**b**), TN (**c**), and TP (**d**) concentrations in the effluent samples and the respective removal efficiencies throughout the operating days. The synthetic influent was prepared with the following concentrations: COD = 500 mg L^−1^, TN = 50 mg L^−1^, NH₄⁺–N = 50 mg L^−1^, and TP = 5 mg L.^−1^

Figure 2a presents the effluent COD concentrations and the corresponding removal efficiencies throughout the entire operational period. COD removal remained consistently above 90% across all aeration conditions, indicating that the decrease in air flow rate from 0.48 to 0.12 m^3^ m^−2^ h^−1^ had no significant impact on COD abatement. Regardless of the DO value in the aerated phase (Table 1), the permeate COD concentrations remained below 50 mg L^−1^, with an average removal efficiency of 97% across all periods (Table 2), indicating no statistically significant differences in COD removal among the evaluated SADₘ conditions (*p* > 0.05).

**Table 2:** Average effluent concentrations and the respective removal efficiency of COD, NH_4_^+^–N, TN, and TP for each experimental period

	COD		NH_4_^+^–N		NO_3_^−^–N		TN		TP	
	Effluent (mg L^−1^)	Removal (%)	Effluent (mg L^−1^)	Removal (%)	Effluent (mg L^−1^)	Removal (%)	Effluent (mg L^−1^)	Removal (%)	Effluent (mg L^−1^)	Removal (%)
Period I	10 ± 6	97.9 ± 1.3	0.2 ± 0.2	99.5 ± 0.5	16.2 ± 3.2	–	9.6 ± 1.2	78.7 ± 3.1	0.46 ± 0.2	91.1 ± 4.3
Period II	12 ± 11	97.7 ± 2.2	2.6 ± 1.6	94.4 ± 3.5	5.7 ± 3.8	–	7.0 ± 2.4	85.1 ± 5.5	0.30 ± 0.3	94.1 ± 5.8
Period III	15 ± 9	97.1 ± 1.8	11.6 ± 7.9	74.7 ± 17.6	4.7 ± 3.2	–	18.5 ± 9.5	59.1 ± 22.1	0.35 ± 0.3	93. 2 ± 4.9

Although permeate quality was not significantly affected, the reduction in aeration led to an increase in the soluble COD concentration in the mixed liquor (Fig. S1, Supplementary Material). As the SADm was progressively reduced to 0.24 m^3^ m^−2^ h^−1^ (period II) and 0.12 m^3^ m^−2^ h^−1^ (period III), the soluble COD (sCOD) concentration at the end of the aerated phase increased by 39% and 43%, respectively (Fig. S2). A mass balance analysis indicated a strong correlation between sCOD and soluble microbial products (“Membrane fouling rate and sludge properties” section), suggesting that the observed increase in sCOD was primarily associated with biopolymer accumulation, rather than a decline in microbial degradation performance. Consequently, membrane filtration assumed a more prominent role in organic matter retention, with its relative contribution to COD removal increasing by 38% and 43.2% in periods II and III, respectively, compared to the observed in period I. This scenario inevitably intensified membrane fouling, as addressed in the “Membrane fouling rate and sludge properties” section.

The negligible impact of decreasing DO on COD removal is also attributed to the effective consumption of organic matter during the non-aerated phase of the SB-EMBR cycle (Fig. S2, Supplementary Material). This operational characteristic enabled the reactor to sustain removal efficiencies above 90%, even with the substantial drop in DO concentration from 6.8 mg L⁻^1^ (period I) to 0.7 mg L⁻^1^ (period III). Thus, the results suggest that operating the SB-EMBR with a non-aerated phase is an effective strategy to maintain COD removal efficiency under limited DO conditions.

### Nitrogen removal

The SB-EMBR exhibited a high ammonium removal performance when operated at a SADm of 0.48 m^3^ m^−2^ h^−1^, with effluent concentration consistently below 1.0 mg L⁻^1^ (Fig. 2b) and average removal efficiency of 99.5% (Table 2). However, as the SADm was progressively reduced to 0.24 and 0.12 m^3^ m^−2^ h^−1^, the nitrification process was adversely impacted, as evidenced by peak NH_4_^+^–N concentrations of 4.9 and 22 mg L⁻^1^ in the SB-EMBR effluent (Fig. 2b). Consequently, the average ammonium removal efficiency declined to 94.4% and 74.7% in these periods, respectively. Thus, the limited DO conditions observed during period III (0.7 ± 0.9 mg L⁻^1^) significantly impaired the ammonium removal performance (*p* < 0.05), whereas the effect observed during period II was not statistically significant (*p* > 0.05).

Despite the oxygen-limiting conditions prevailing during period III, a decreasing trend in effluent NH_4_^+^–N concentrations was observed from operational day 122 onwards. By day 150, the ammonium concentration in the effluent had decreased to 3 mg L⁻^1^, corresponding to a removal efficiency of 94.2%. This behavior may suggest a potential acclimation of the nitrifying community to oxygen-limited conditions, as reported in previous studies (Fan et al. 2017). The enhanced nitrification performance observed toward the end of period III may be attributed to the enrichment of ammonia-oxidizing (AOB) and nitrite-oxidizing bacteria (NOB) strains capable of withstanding low DO conditions. According to Park and Noguera (2004), extended operation under oxygen-limited environments promoted the emergence of distinct subgroups within the *Nitrosomonas europaea* lineage, which supported stable nitrification activity even under reduced oxygen availability. In line with these findings, Li et al. (2018) reported that low dissolved oxygen levels can favor the selection of specialized nitrifiers adapted to suboptimal oxygen conditions. In their study, *Nitrosomonas*-like AOB populations demonstrated stable activity at DO concentrations as low as 0.5 mg L⁻^1^. The authors also highlighted that prolonged low DO exposure can shift the microbial community structure toward organisms with enhanced oxygen affinity, supporting ammonia oxidation even in oxygen-limiting environments. Such microbial adaptation mechanisms may partially explain the recovery in ammonium removal observed in the latter part of period III in the present study.

**Fig. 3:**
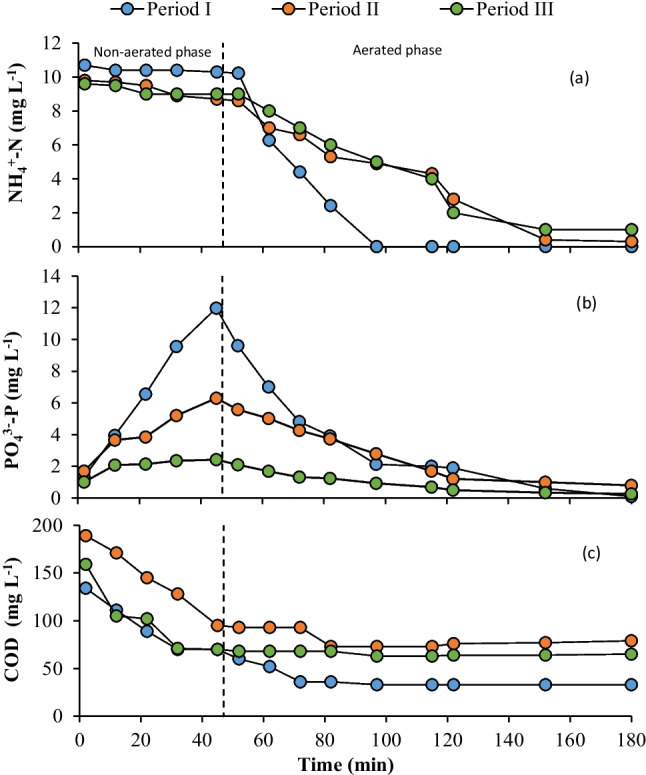
**a** NH_4_^+^–N, **b** PO_4_^3−^–P, and **c** COD concentration profiles during the non-aerated and aerated phases of the SB-EMBR cycle at the end of each experimental period

Results from the SB-EMBR cycle analysis (Fig. 3a) revealed a pronounced decline in the specific ammonium uptake rate (SAUR) as aeration intensity decreased during periods II and III. SAUR values dropped from 3.58 mg NH_4_^+^–N g⁻^1^ MLVSS h⁻^1^ in period I to 1.16 and 0.97 mg NH_4_^+^–N g⁻^1^ MLVSS h⁻^1^, corresponding to reductions of 67% and 72%, respectively. This trend aligns with the decreased NH_4_^+^–N removal efficiencies observed under lower DO concentrations (Fig. 2b), indicating a suppression of nitrifying activity. The most substantial decline occurred under the lowest aeration rate (SADₘ = 0.12 m^3^ m⁻^2^ h⁻^1^), where DO levels consistently remained below 1 mg L⁻^1^. In oxygen-limited conditions (DO < 1.5 mg L⁻^1^), Li et al. reported a substantial decline in SAUR, while rates around 5.5 mg NH₄⁺–N g⁻^1^ VSS h⁻^1^ were maintained when DO was above 2.5 mg L⁻^1^ (Li et al. 2018). These results reinforce the strong dependency of SAUR on oxygen availability and highlight the detrimental effect of oxygen-limiting conditions on nitrifier metabolism. The low SAUR values observed under reduced DO highlight the importance of maintaining adequate aeration. However, the application of high SADₘ values should be carefully evaluated, as the optimal aeration condition depends on multiple factors, including effluent quality requirements, energy efficiency, and membrane fouling control in SB-EMBR systems.

Besides not exerting a significant adverse effect on NH_4_⁺–N removal, the reduction of SADm in period II contributed to an improvement in TN removal. When the aeration intensity was reduced to 0.24 m^3^ m⁻^2^ h⁻^1^, the average TN removal efficiency increased from 78.7% (period I) to 85.1% (period II) (Table 2). This enhancement may be attributed to the occurrence of simultaneous nitrification and denitrification (SND), wherein differential diffusion of DO across sludge flocs creates coexisting aerobic and anoxic microenvironments (Chang et al. 2022). Under such conditions, aerobic zones near the floc surface support nitrification, while anoxic regions in the floc core enable denitrification to proceed simultaneously. Huang et al. (2022) reported that SND can occur within a DO concentration range of 0.5 to 3 mg L–^1^, with optimal performance observed between 1 and 2 mg L^–1^. Accordingly, the observed decrease in DO concentration to 2.7 ± 0.7 mg L⁻^1^ (Table 1) during period II likely favored TN removal via the SND pathway.

On the other hand, TN removal performance declined substantially when the SB-EMBR was subjected to a further reduction in the SAD_m_ to 0.12 m^3^ m^−2^ h^−1^. As shown in Fig. 2c, the TN concentration in the permeate abruptly increased, reaching a peak of 32 mg L⁻^1^ and a corresponding removal efficiency of only 28%. Over the subsequent days, TN removal remained highly unstable, indicating a deterioration of both nitrification and denitrification processes under oxygen-limited conditions (DO = 0.7 ± 0.9 mg L⁻^1^). Nevertheless, from operational day 122 onwards, as NH_4_⁺–N removal progressively improved (Fig. 2b), TN removal became more stable and consistent, with efficiencies exceeding 80% in the final part of period III.

### Phosphorus removal

The SB-EMBR exhibited stable and consistent TP removal performance throughout the three operational periods. Regardless of the aeration intensity applied, effluent TP concentrations remained below 1 mg L^−1^ (Fig. 2d), with average removal efficiencies exceeding 90% (Table 2), indicating that TP removal was not significantly affected by the evaluated SADₘ conditions (*p* > 0.05). These results indicate that phosphorus removal was not significantly affected by the reduction in aeration intensity from 0.48 to 0.12 m^3^ m^−2^ h^−1^. This stability can be attributed to the electrocoagulation process, which provided highly effective phosphorus removal independent of aeration intensity. Based on the estimated anodic dissolution rate (*W* = 10.1 g Al^3^⁺ m⁻^3^; “Anode dissolution and energy consumption” section) and the influent TP concentration (5 mg L⁻^1^), the theoretical molar Al/P ratio was calculated as 2.32 mol Al mol^−1^ P. This ratio is stoichiometrically sufficient to precipitate phosphate as AlPO_4_ (1:1 molar requirement) and also to generate amorphous Al(OH)_3_ flocs capable of adsorbing residual phosphate species (Giwa et al. 2016; Souza et al. 2020). Therefore, the consistently low effluent TP concentrations indicate that electrocoagulation alone provided sufficiently favorable conditions for phosphorus removal through physicochemical pathways. The anodically generated Al^3^⁺ ions and hydroxide flocs ensured efficient phosphate precipitation and adsorption, effectively sustaining high TP removal efficiency even under the reduced aeration intensities applied in periods II and III, when biological phosphorus uptake could have been partially suppressed.

To further clarify the impact of aeration intensity on enhanced biological phosphorus removal (EBPR), reactor cycle analyses were conducted during each experimental period to assess the maximum P-release and P-uptake activities. The results revealed a substantial decline in both P-release and P-uptake values as the aeration intensity was reduced in periods II and III (Fig. 3b). Compared to the value obtained in period I (14.89 mg P L⁻^1^ h⁻^1^), the calculated P-release rate (PRR) decreased by 57% (6.42 mg P L⁻^1^ h⁻^1^) and 86% (1.97 mg P L⁻^1^ h⁻^1^) in the periods II and II, respectively (Table 3). A similar trend was observed for the P-uptake rate (PUR) as the aeration intensity decreased (Table 3). These findings clearly indicate a deterioration of the EBPR process under oxygen-limited conditions.

**Table 3:** Volumetric and specific phosphorus release and uptake rates, and P-release-to-COD uptake ratios during each operational period

	P-release			P-uptake	
	PRR (mgP L^−1^ h^−1^)	SPRR (mgP gMLVSS^−1^ h^−1^)	P_release_/COD_uptake_ ratio (mol-P/mol-C)	PUR (mgP L^−1^ h^−1^)	SPUR (mgP gMLVSS^−1^ h^−1^)
Period I	14.89	4.17	0.172	11.40	3.18
Period II	6.42	1.90	0.056	4.10	1.20
Period III	1.97	0.55	0.0164	1.70	0.48

*PRR*, P-release rate; *SPRR*, specific P-release rate; *PUR*, P-uptake rate; *SPUR*, specific P-uptake rate

The PUR behavior observed in the present study contrasts with the results reported by Belli et al. (2021), who observed a substantial increment in the PUR value when the aeration intensity was reduced from 0.426 to 0.106 m^3^ m⁻^2^ h⁻^1^ in a non-electrochemically assisted SB-MBR. The conflicting results may be attributed to the continuous use of electrocoagulation in the present study, which likely imposed inhibitory effects on the polyphosphate-accumulating organisms (PAOs). Previous studies have shown that chemical coagulants such as ferric chloride (FeCl₃) and polyaluminum chloride (PAC) can impair both P-release and P-uptake processes (Yang et al. 2019; Li et al. 2021). In this context, the pronounced reduction in PRR and PUR values throughout the experimental period may be linked to PAOs inhibition caused by the in situ generation of coagulants via electrochemical anode dissolution. When investigating the long-term effect of coagulants addition on EBPR process, Xia et al. (2022) observed a decrease in the relative abundance of *Ca. Accumulibacter*, a key polyphosphate-accumulating organism, following the addition of iron- and aluminum-based salts. This microbial suppression negatively affected the TP removal, which was partially reversed once metal salt dosing was discontinued.

The ratio between the phosphate release rate and the COD uptake rate during the anaerobic phase provides valuable insight into EBPR performance (Schuler and Jenkins 2003). Previous studies have indicated that a P-release/COD-uptake ratio of 0.5 or higher is typically associated with polyphosphate-accumulating metabolism as the dominant pathway for carbon uptake in EBPR processes (Zhang et al. 2025). In the present study, the calculated P-release/COD-uptake ratio decreased markedly from 0.172 to 0.0164 mol-P/mol-C over the three experimental periods (Table 3), indicating a substantial decoupling between phosphate release and carbon uptake and suggesting progressive suppression of EBPR activity in the SB-EMBR system.

Despite the marked reduction in the P-release/COD-uptake ratio, effluent TP concentrations remained below 1 mg L⁻^1^ throughout all operational periods, with removal efficiencies consistently exceeding 90%. This behavior indicates that the reductions in the P-release/COD-uptake ratio, as well as in the PRR and PUR values (Table 3), did not compromise the overall TP removal performance of the reactor. Under these conditions, it is reasonable to assume that phosphorus removal through physicochemical mechanisms associated with electrocoagulation became progressively predominant during periods II and III as the EBPR process lost performance. Further investigations employing molecular or sequencing-based techniques could provide deeper insights into the dynamics of phosphate-accumulating organisms and the long-term impacts of electrocoagulation on EBPR activity.

Thus, in MBR systems assisted by electrochemical processes, long-term operation involving electrocoagulation may adversely affect the EBPR process, reducing the contribution of biological phosphorus removal while increasing the contribution of physicochemical removal pathways. To mitigate these impacts and ensure effective simultaneous chemical and biological phosphorus removal, periodic interruption of the electrocoagulation process should be considered.

**Fig. 4:**
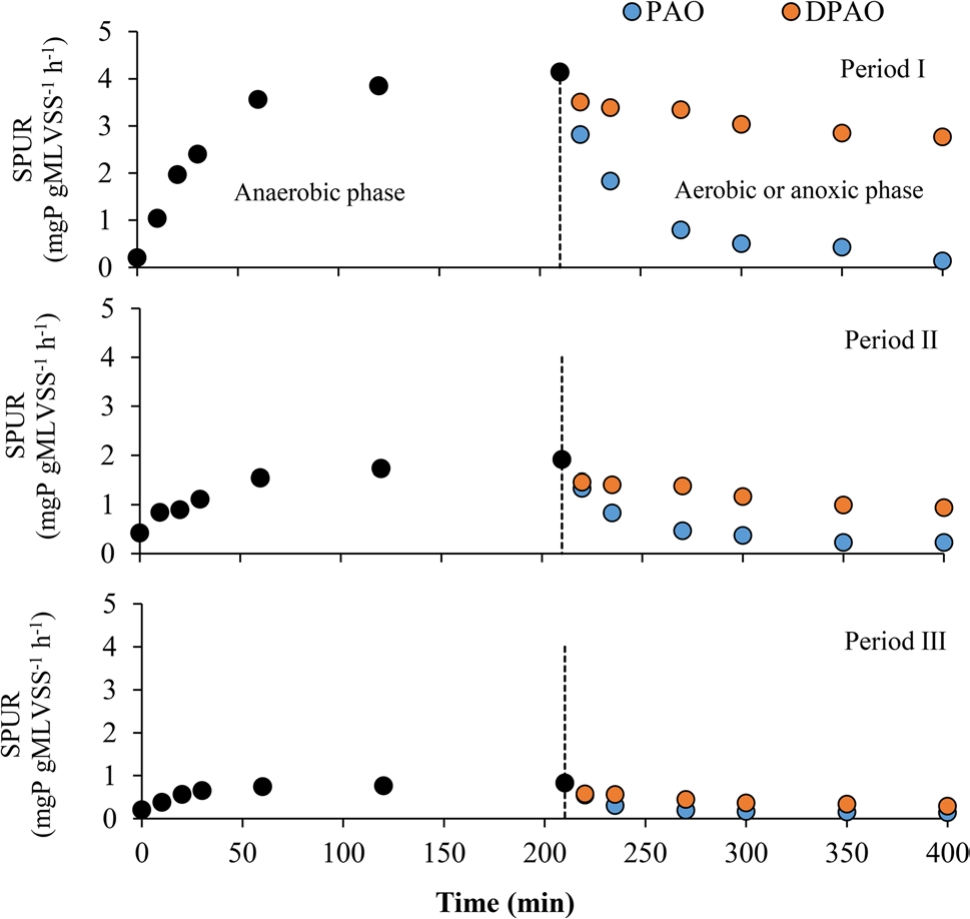
Aerobic and anoxic phosphate uptake potential obtained from batch tests conducted during each experimental period

Batch tests were conducted using biomass from the SB-EMBR to evaluate the aerobic and anoxic P-uptake potential under each applied aeration intensity (Fig. 4). The results confirmed a substantial decline in the aerobic P-uptake during periods II and III, which is consistent with the behavior previously observed in the reactor cycle analyses. In contrast, the anoxic P-uptake potential exhibited a relative improvement as the aeration intensity decreased (Table S1). Although absolute anoxic uptake rates declined, the proportion of phosphate uptake occurring under anoxic conditions, expressed as the denitrifying dephosphatation potential (DDP), increased from 18% in period I to 41% in period III. These results suggest a shift in PAOs metabolism toward enhanced utilization of nitrate as an electron acceptor under oxygen-limited conditions. Maximizing anoxic P-uptake offers several operational advantages, as it enables the simultaneous removal of nitrate and phosphate using the same carbon source, thereby enhancing overall nutrient removal efficiency (Monclús et al. 2010). Additionally, this pathway reduces aeration requirements due to the use of nitrate, instead of oxygen, as the terminal electron acceptor during intracellular polyhydroxyalkanoate (PHA) degradation in polyphosphate-accumulating organism-enriched systems (Costa et al. 2017). Such characteristics are particularly beneficial for improving energy efficiency and sustainability in wastewater treatment processes operating under low DO conditions.

### Membrane fouling rate and sludge properties

**Fig. 5:**
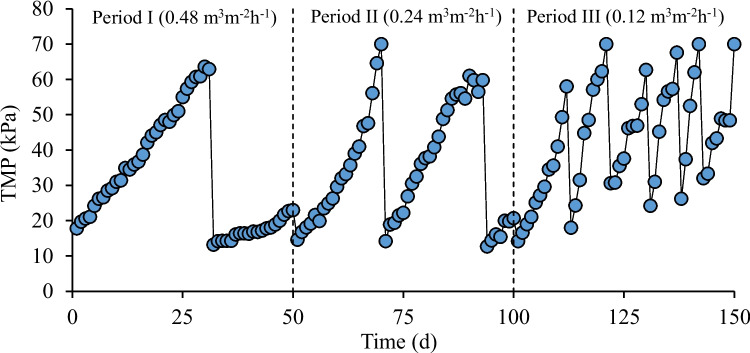
Transmembrane pressure values throughout the three experimental periods

A more pronounced increase in transmembrane pressure (TMP) was observed as the SADₘ was diminished from periods I (0.48 m^3^m^−2^ h^−1^) to III (0.12 m^3^m^−2^ h^−1^) (Fig. 5). Consequently, the membrane fouling rate (MFR) intensified with the reduction in aeration intensity, reaching average values of 1.02, 2.69, and 4.81 kPa day⁻^1^ during periods I, II, and III, respectively. Based on TMP values measured before and after membrane cleaning procedures, the membrane resistance attributed to reversible (${R}_{r}$) and irreversible fouling (${R}_{ir}$) were calculated. The results indicated that reversible fouling remained the predominant fraction throughout reactor operation, with ${R}_{r}$ values ranging from 8.73 × 10^12^ to 1.07 × 10^13^ m^−1^ (Table 4). Nevertheless, ${R}_{ir}$ increased substantially during period III, rising from approximately 1.4 to 1.5 × 10^12^ m^−1^ in periods I and II to 3.40 × 10^12^ m^−1^ in period III. This behavior indicates that aeration reduction favored the formation of a more compact and less removable fouling layer. Similar behavior has been reported in MBR systems subjected to changes in aeration-induced shear, in which shifts toward smaller and more compact flocs promoted the formation of less permeable cake layers and intensified irreversible fouling through pore blocking mechanisms (De Temmerman et al. 2015).

**Table 4:** Average values of parameters related to membrane fouling propensity in the SB-EMBR mixed liquor

	Units	Period I	Period II	Period III
SAD_m_	m^3^ m^−2^ h^−1^	0.48	0.24	0.12
MFR	kPa d^−1^	1.02 ± 0.67	2.69 ± 0.32	4.81 ± 1.11
R_r_	m^−1^	1.02 × 10^13^	1.07 × 10^13^	8.73 × 10^12^
R_ir_	m^−1^	1.44 × 10^12^	1.49 × 10^12^	3.40 × 10^12^
SRF	m kg^−1^	2.60 × 10^13^	3.62 × 10^13^	4.22 × 10^13^
CST	s	22.1 ± 5.1	30.2 ± 4.1	49.5 ± 9.2
PSD	μm	69.2.4 ± 8.2	41.3 ± 8.8	34.5 ± 1.5
SNT	NTU	24.9 ± 5.4	85.3 ± 2.4	93.8 ± 17.1
MLSS	mg L^−1^	4530 ± 160	4790 ± 350	5140 ± 510
MLVSS	mg L^−1^	3460 ± 370	3760 ± 250	3900 ± 400
MLSS/MLVSS	-	0.76	0.79	0.76

*SAD*_*m*_, specific aeration demand per membrane area; *MFR*, membrane fouling rate; *R*_*r*_, reversible fouling resistance; *R*_*ir*_, irreversible fouling resistance; S*RF*, specific resistance to filtration; *CST*, capillary suction time; *PSD*, particle size distribution; *SNT*, supernatant turbidity; *MLSS*, mixed liquor suspended solids; *MLVSS*, mixed liquor volatile suspended solids

Consistent with the progressive increase in MFR, the dead-end filtration tests also revealed a marked increase in specific resistance to filtration (SRF) as the SADₘ decreased. SRF values increased from 2.60 × 10^13^ m kg^−1^ in period I to 3.62 × 10^13^ and 4.22 × 10^13^ m kg^−1^ in periods II and III, respectively (Table 4), indicating reduced mixed liquor filterability under oxygen-limited conditions.

The increase in MFR observed in the SB-EMBR system can be primarily attributed to the diminished shear forces and reduced air scouring under lower SADₘ conditions, which likely favored the adhesion of biofoulants to the membrane surface, thereby intensifying membrane fouling (Judd 2006). Furthermore, the reduction in aeration led to an increase in soluble organic content in the mixed liquor, as indicated by the elevated sCOD concentrations observed during periods II and III (Fig. S2). The accumulation of high-molecular-weight compounds in the supernatant may have further contributed to membrane fouling. Wu and Huang (2009) observed a strong correlation between supernatant organic content and membrane filterability (*R* = 0.804), highlighting their relevance as dominant foulants in MBR systems. Since this organic content is mainly composed of SMPs, predominantly proteins and polysaccharides, a theoretical estimation of their contribution to sCOD was carried out. Conversion factors of 1.2 g COD per g of protein and 1.13 g COD per g of polysaccharides were applied (HeNZE 2002). A positive correlation (*R*^2^ = 0.6152) was found between the measured sCOD at the end of the aerated phase and the estimated COD from SMP (Fig. S3), supporting the hypothesis that the increase in sCOD under reduced aeration was largely associated with SMP accumulation. Despite this, no deterioration in permeate quality was observed, as discussed in the “Chemical oxygen demand (COD) removal” section. This finding highlights the critical role of the ultrafiltration membrane in retaining macromolecules with high molecular weight, thereby preventing deterioration in permeate quality.

Despite the progressive increase in MFR with decreasing SADm, the values observed in this study remained considerably lower than those typically reported for non-electrochemically assisted SB-MBRs and operated under comparable aeration regimes. For instance, Belli et al. (2021) reported an MFR of 2.89 kPa d⁻^1^ in a conventional SB-MBR operated at a SAD_m_ of 0.426 m^3^ m⁻^2^ h⁻^1^, which is approximately 183% higher than the value observed in the present study for a comparable SAD_m_ (Table 4). The integration of electrochemical processes into MBRs has been increasingly recognized as a promising strategy to alleviate membrane fouling (Zhang et al. 2023). Souza et al. (2020) reported a 65% decrease in MFR (from 3.42 to 1.19 kPa day⁻^1^) upon applying a current density of 10 A m⁻^2^ in an SB-EMBR system. The effectiveness of electrocoagulation in fouling control is primarily attributed to electrochemically induced changes in sludge properties, including decreased concentrations of SMP, enlarged floc size, and decreased levels of soluble residual COD (Borea et al. 2019). In this scenario, the filterability of the mixed liquor is improved, and the fouling propensity is consequently reduced.

The reduction in membrane fouling observed in electrochemically assisted MBRs is not exclusively due to the coagulation effects. Other electrochemical processes may also occur simultaneously in the mixed liquor during the application of an electric field. Among these, electrophoresis can enhance the repulsion between foulants and the membrane surface, which may lead to the formation of a loosely attached cake layer (Ho et al. 2017). Electroosmosis may also take place and facilitate the release of bound water from microbial flocs, a property that significantly influences sludge filterability (Ibeid et al. 2013). Thus, the control of membrane fouling in EMBR systems may be attributed to a combination of electrocoagulation, electrostatic repulsion of foulants, and improved biomass dewatering.

Fouling-related parameters monitored in the present study indicated a deterioration in mixed liquor filterability as aeration intensity was reduced (Table 4). The progressive increase in average CST values during periods II and III reflected a decline in sludge dewaterability, suggesting an elevated membrane fouling potential under reduced aeration conditions (Abu-Obaid et al. 2020). Simultaneously, the observed reduction in particle size distribution (PSD) values pointed to a shift toward smaller flocs at lower dissolved oxygen concentrations. This trend aligns with previous findings by Faust et al., who reported significantly smaller average floc sizes in a conventional MBR operated at 1 mg L^−1^ DO compared to 4 mg L^−1^ (Faust et al. 2014). Similar to the present study, those authors also observed an increase in supernatant turbidity (SNT) under oxygen-limited conditions, reinforcing that the reduced aeration intensity applied during periods II and III impaired the bioflocculation process, which in turn contributed to the increase in membrane fouling rates during these periods.

**Fig. 6:**
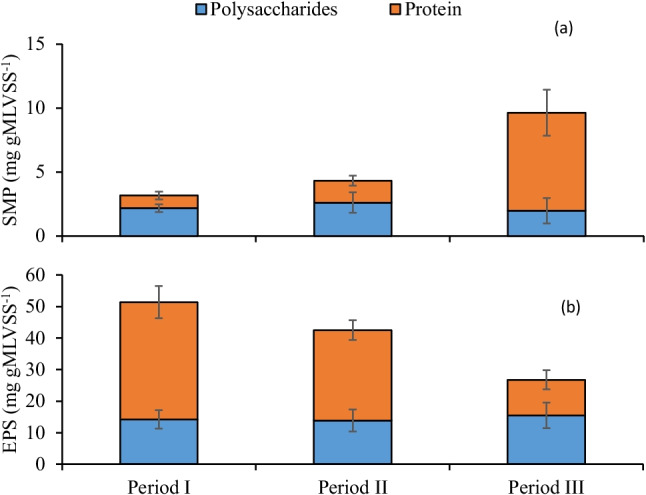
Average concentrations of SMP (**a**) and EPS (**b**) in terms of proteins and polysaccharides in the periods I, II, and III

The reduction in floc size and elevated SNT at low oxygen concentrations may be associated with a lower production of extracellular polymeric substances (EPS), which play a key role in maintaining floc integrity and promoting particle aggregation. As a result of the deteriorated bioflocculation process, fine particles remained dispersed in the mixed liquor, adversely affecting membrane permeability. In fact, the EPS concentration decreased while the SMP increased as the aeration intensity was diminished (Fig. 6). From periods I to III, the EPS content decreased by 48% whereas the SMP increase by 205%. At same time, the proportion of proteins in relation to the total EPS reduced from 73% (37.1 mg gMLVSS^−1^) to 42% (11.3 mg gMLVSS^−1^), while the polysaccharides content remained practically constant (14.2 to 15.5 mg gMLVSS^−1^). These results indicate that oxygen-limited conditions mainly impacted the protein fraction of the total EPS content. This is particularly relevant given that EPS proteins are recognized as the key contributors to bioflocculation, mainly due to their hydrophobic nature and ability to promote microbial aggregation through electrostatic interactions (Chen et al. 2010; Emaminejad et al. 2019). A strong positive correlation between EPS proteins and bioflocculation efficiency was previously reported (Emaminejad et al. 2019), highlighting their critical role in forming stable flocs. Therefore, the observed reduction in EPS protein content likely impaired sludge floc integrity and cohesion, contributing to the decrease in floc size (lower PSD) and the increase in supernatant turbidity under reduced aeration. These changes ultimately compromise mixed liquor filterability and intensify membrane fouling under oxygen-limited regimes.

Overall, the combined evaluation of CST, particle size distribution, SRF, and EPS/SMP profiles provided consistent evidence explaining the progressive increase in membrane fouling under lower SADₘ conditions, indicating a marked deterioration in mixed liquor filterability throughout the operational periods. As demonstrated by Galizia et al. (2025), decreasing SADₘ below critical thresholds resulted in a marked increase in fouling rates, even under subcritical flux conditions. Their aeration-step tests further confirmed that reduced aeration compromised fouling control, and irreversible fouling occurred even after restoring SAD_m_, highlighting the importance of maintaining aeration within an optimal operational range to mitigate the fouling process. On the other hand, excessively high aeration rates do not necessarily mitigate fouling and may also induce adverse effects. As evidenced by Meng et al., elevated air flow rates promoted sludge floc disintegration, resulting in the release of colloids and solutes into the bulk liquid, which negatively affected membrane permeability (Meng et al. 2008). These results suggest the existence of a critical aeration threshold, outside of which fouling propensity increases. Accordingly, the selection of an appropriate SAD_m_ should consider the trade-off between membrane fouling control and energy efficiency and must be optimized in accordance with the specific membrane configuration and operational conditions of the MBR system.

Although the set of analyses performed in the present study provided consistent evidence supporting the observed fouling trends, future investigations employing additional approaches, such as fouling layer characterization, resistance-in-series analysis, and reversible/irreversible fouling assessment, could further improve the understanding of membrane fouling phenomena under reduced SADₘ conditions in SB-EMBR systems.

### Anode dissolution and energy consumption

Taking into account the daily wastewater flow and the anode dissolution rate (m) determined from Eq. 3, the corresponding volumetric anode corrosion rate (W) was estimated at 10.1 g Al^3+^ m^−3^. This value is substantially lower than those reported for EMBR systems operated under continuous-flow conditions (Table 5). For instance, Udomkittayachai et al. (Udomkittayachai et al. 2021) reported an anodic corrosion rate of 35 g Al^3+^ m^−3^ during municipal wastewater treatment using a continuous EMBR configuration operated at a current density of 15 A m^−2^.

**Table 5:** Parameters related to anodic corrosion, energy consumption, and additional operating costs associated with the electrocoagulation process

Reference	Influent type	Mode	T_EC_ (h d^−1^)	CD (A m^−2^)	W (g Al^3+^ m^−3^)	EC (kWh m^−3^)	C (USD m^−3^)	q (mAh L^−1^)
This study	Synthetic wastewater	Sequencing batch	1.6	10	13.4	0.37	0.07	39.9
Ravadelli et al. (2021)	Textile wastewater	Continuous	4	10	41.4	1.07	0.19	139.6
Mendes Predolin et al. (2021)	Municipal wastewater	Continuous	8	10	–	0.86	–	428.3
Sharghi et al. (2024)	Refinery wastewater	Continuous	6	14	145	0.43	0.90	214.7
Farsani et al. (2022)	Landfill leachate	Continuous	6	5	72	1.57	0.167	90.6

*T*_*EC*_, current application time; *I*, electric current; *m*, anodic dissolution rate per time; *W*, anodic dissolution rate per volume of permeate; *EC*, energy consumption; *C*, additional operating cost; *q*, electric charge loading

The lower corrosion rate observed in the present study can be attributed to the limited electric current application time (*T*_EC_) in the SB-EMBR. Unlike continuous-flow EMBRs, where current is applied continuously (albeit intermittently), the SB-EMBR was exposed to electric current only during the aeration/filtration phase (65 min). As a result, the total daily current application in the SB-EMBR was limited to 1.6 h, whereas in continuous systems, values ranging from 4 to 12 h day^−1^ have been reported (Table 5).

This operational feature also contributed to a lower energy consumption (EC) associated with electrocoagulation, which was estimated at 0.37 kWh m^−3^. This value is approximately 2.3 times lower than that reported by Mendes Predolin et al. (2021) for a continuous-flow EMBR treating municipal wastewater. These findings indicate that operating the EMBR in sequencing batch mode offers energy-saving advantages.

Based on the calculated EC and *W* values, the additional operating cost (*C*) related to electrocoagulation in the SB-EMBR was estimated at 0.07 USD m^−3^. This value is markedly lower than those reported for continuous EMBRs treating both municipal and industrial wastewaters (Table 5). When compared to conventional MBRs, the cost of 0.07 USD m⁻^3^ corresponds to 49% of the operating cost estimated for full-scale MBRs treating municipal wastewater, which is approximately 0.15 USD m^−3^ (Gao et al. 2021). Although the integration of electrocoagulation into MBR systems may increase operational costs, this limitation can be mitigated by the decreased need for membrane cleaning, attributed to the lower fouling rates typically reported for EMBRs (Asif et al. 2020). Xiao et al. for example, estimated that membrane cleaning chemicals alone can account for 10–30% of the total operating cost of an MBR (Xiao et al. 2019). Furthermore, the incorporation of electrocoagulation has been associated with improved permeate quality, which is particularly advantageous for applications involving water reuse.

According to Eq. 6, the volumetric charge loading (*q*) was estimated at 39.9 mAh L⁻^1^. This value is markedly lower than those reported in previous studies, where charge loadings ranged from 90.6 to 428.3 mAh L⁻^1^ (Table 5). These results highlight the potential of the SB-EMBR to achieve high pollutant removal efficiencies while simultaneously mitigating membrane fouling. The reduced volumetric charge loading observed in this study demonstrates the enhanced process performance and cost-effectiveness of the SB-EMBR, reinforcing its advantage over EMBRs operated in continuous-flow mode.

In addition to the energy demand associated with the electrocoagulation process, the energy consumption related to aeration is also an important operational aspect in SB-EMBR systems, since aeration is typically recognized as one of the major contributors to the overall energy consumption in MBR systems. Therefore, an estimative of the aeration energy demand was also performed for the SB-EMBR operation. The estimation was based on the nominal power consumption of the air compressor (THOMAS, LP-150HN), the daily treated wastewater volume (129.6 L day^−1^), and the cumulative aeration time applied throughout the sequential batch operational cycles (18 h day^−1^). Based on these operational conditions, the estimated aeration energy demand for period I was 18.06 kWhm^−3^. For comparative purposes, the aeration energy demand in periods II and III was estimated proportionally to the reduction in SADₘ, resulting in estimated consumptions of 9.03 and 4.52 kWhm^−3^, respectively. It should be noted that the estimated energy values obtained in the present study refer to a bench-scale system and are therefore higher than those typically reported for full-scale MBR systems due to scale limitations and the lower energy efficiency of laboratory equipment. Recent long-term full-scale evaluations of industrial MBR systems have reported total electricity demands ranging from 0.37 to 0.82 kWh m^−3^, with aeration accounting for approximately 45–75% of the total plant energy demand (Zulhendri et al. 2026).

## Conclusions

The performance of a sequencing batch electro-membrane bioreactor (SB-EMBR) operated under low electric charge loading (39.9 mAh L⁻^1^) and different aeration intensity was evaluated. Reducing the specific aeration demand (SADₘ) from 0.48 to 0.12 m^3^ m⁻^2^ h⁻^1^ had negligible impact on COD and phosphorus removal, with average COD removal efficiencies exceeding 97% and permeate TP concentrations consistently below 1 mg L⁻^1^. The consistently high COD removal was partly attributed to the membrane ability to retain soluble microbial products, whose accumulation under reduced aeration conditions contributed to elevated sCOD concentrations in the mixed liquor. In contrast, ammonium removal was adversely affected at lower SADₘ, particularly at 0.12 m^3^ m⁻^2^ h⁻^1^, when the average efficiency decreased to 74.7%. Nevertheless, a progressive recovery of ammonium oxidation process was observed, achieving over 90% NH_4_^+^–N removal by the end of the operational period. Reactor cycle analyses confirmed that nitrifying activity was impaired under reduced aeration, as evidenced by the decrease in the specific ammonium uptake rate from 3.58 to 1.16 and 0.97 mg NH_4_^+^–N g⁻^1^ MLVSS h⁻^1^. Similar trends were observed in the P-release and P-uptake activities, indicating a deterioration of biological phosphorus removal under oxygen-limited conditions. The substantial decrease in the P-release/COD-uptake ratio from 0.172 to 0.0164 mol P mol⁻^1^ C suggests that physicochemical phosphorus removal associated with electrocoagulation became progressively more relevant as biological phosphorus removal activity declined. Interestingly, a higher anoxic P-uptake potential at lower aeration intensities was observed, a promising finding in terms of aeration-related energy savings. Despite this potential advantage, lower airflow rates negatively impacted mixed liquor filterability, as reflected by several fouling-related parameters. Although the membrane fouling rate increased under reduced SADₘ (1.02–4.81 kPa day⁻^1^), the values remained consistently lower than those typically reported for non-electrochemically assisted membrane bioreactors. Due to the short period of electric current application (1.6 h day⁻^1^), the estimated additional electrocoagulation cost was only 0.07 USD m⁻^3^, substantially lower than those reported for continuous-flow electro-membrane bioreactors. These findings demonstrate that operating the SB-EMBR under reduced electric charge loading is a feasible strategy to minimize energy demand while maintaining satisfactory pollutant removal even under oxygen-limited conditions, although reduced SADₘ negatively affected nitrification performance and membrane fouling behavior.

## Supplementary Information

Below is the link to the electronic supplementary material.

ESM1 (DOCX 43.9 KB)

## Data Availability

All data supporting this study are available.
