# Supplementary material for: Low electric charge loading in a sequencing batch electro-membrane bioreactor: influence of aeration intensity on treatment performance, biomass activity, and membrane fouling
Source: Environ Sci Pollut Res Int. 2026 Jul 1;33(21):10483–98. doi: 10.1007/s11356-026-37984-6 (PMC13368971; doi:10.1007/s11356-026-37984-6)
Supplement: Supplementary file 1 — (DOCX 43.9 KB) [file 11356_2026_37984_MOESM1_ESM.docx]

**Supplementary Material: Low electric charge loading in a sequencing batch electro-membrane bioreactor: influence of aeration intensity on treatment performance, biomass activity and membrane fouling.**

Figure S1: Average soluble COD (sCOD) at the end of aerated phase and in the permeate and the corresponding sCOD removed by the membrane during each experimental periods.

Fig. S2 – Average COD consumption in the non-aerated and aerated phases of the SB-EMBR operating cycle throughout each experimental period.

Fig. S3 - Correlation between measured soluble COD (sCOD) at the end of the aerated phase and the theoretical COD linked to soluble microbial products (SMPs).

Table S1 - Specific phosphate uptake rate (SPUR) under aerobic and anoxic conditions and the corresponding denitrifying dephosphatation potential for each period.

|  | Aerobic  (mgP gMLVSS^-1^ h^-1^) | Anoxic  (mgP gMLVSS^-1^ h^-1^) | DDP^1^  (%) |
| --- | --- | --- | --- |
| Period I | 3.35 | 0.74 | 18 |
| Period II | 1.03 | 0.39 | 28 |
| Period III | 0.44 | 0.31 | 41 |

^1^DDP: denitrifying dephosphatation potential
